# Bio-control on the contamination of Ochratoxin A in food: Current research and future prospects

**DOI:** 10.1016/j.crfs.2022.09.007

**Published:** 2022-09-11

**Authors:** Leran Wang, Qi Wang, Saiqun Wang, Rui Cai, Yahong Yuan, Tianli Yue, Zhouli Wang

**Affiliations:** a College of Food Science and Engineering, Northwest A&F University, Yangling, Shaanxi, 712100, China; b Laboratory of Quality & Safety Risk Assessment for Agro-products (YangLing), Ministry of Agriculture, Yangling, Shaanxi, 712100, China; c College of Food Science and Engineering, Northwest University, Xi'an, Shaanxi, 710069, China

**Keywords:** Ochratoxin A, Biological methods, Degradation, Adsorption, Metabolic pathways

## Abstract

Ochratoxin A (OTA) is a secondary metabolite of several fungi and widely exists in various species of foods. The establishment of effective methods for OTA reduction is a key measure to ensure food processing and human health. This article reviews the current research of OTA reduction by biological approaches, summarizes the characteristics and efficiency of them, and evaluates the transformation pathways and metabolites safety of each degradation technology. The shortcomings of various methods are pointed out and future prospects are also proposed. Biological methods are the most promising approaches for OTA control. The defect of them is the long processing time and the growth of microbial cells may affect the product quality. Therefore, the control of OTA contamination should be conducted according to the food processing and their product types. Besides, it is significant for the exploitation of new strains, enzyme and novel adsorbents.

## Highlights

• The application of physical and chemical methods has been restricted.
• Existing biological methods can effectively detoxify OTA.
• OTA reduction systems should be established for different food.
• The exploitation of novel equipment, enzyme and adsorbents is essential.

## Graphical abstract

**Figure:**
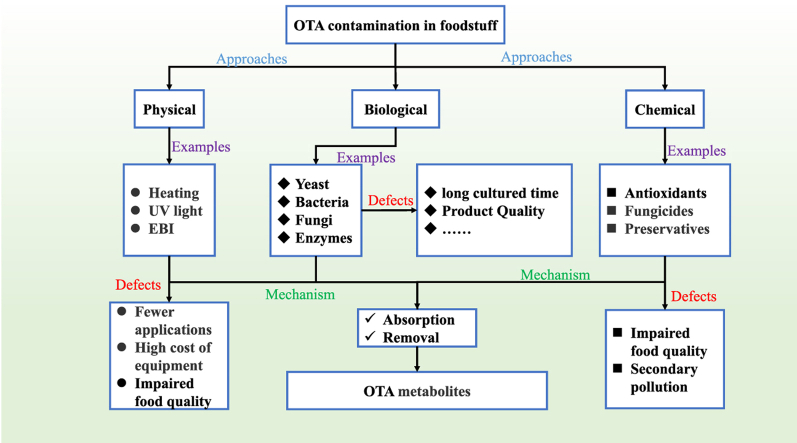

## 1 Introduction

Ochratoxin A (OTA) is a secondary metabolite produced by several fungi of *Aspergillus* and *Penicillium* (Bryła et al., 2021; Chen et al., 2018; Gu et al., 2021). The chemical structure of OTA consists of a 7-carboxy- 5-chloro -8-hydroxy-3, 4-dihydro- 3- methylcoumarin, linked through the 7-carboxy- group to L-β- phenylalanine by an amide bond. As one of the most important mycotoxins concern for human health, OTA exhibits nephrotoxic, mutagenic, immunotoxic, teratogenic and carcinogenic properties (Cuciureanu et al., 2021; Hashemi et al., 2021). For a long time, the contamination of OTA has been monitored in a variety of foods, such as meat, eggs, fruit and vegetables, dairy products, milk-based baby formulae and infant cereals. In particular, for the growth processes are susceptible to fungal infection, grains, coffee, grapes and their products are the most easily contaminated food (Cebin Coppa, Khaneghah, Alvito, Assuncao, Martins, Es, et al., 2019; Khaneghah et al., 2019; Misihairabgwi et al., 2019). The presence of OTA in healthy human blood confirms widespread and continuous exposure. For the various contamination levels of OTA in different regions and products, the maximum levels of OTA may vary considerably. Codex Alimentarius Commission (CAC) has set the OTA limit in wheat, barley and rye is 5 μg/kg and there is no limit for their processed products. International Vine and Wine Organization (OIV) has presented the OTA limit in wine is 2 μg/kg. The European Commission establishes the maximum permitted levels of OTA for grape juice and wine (2 μg/kg), raw cereal grains (5 μg/kg) and cereals products (3 μg/kg), roasted coffee (5 μg/kg) and instant coffee (10 μg/kg), dried fruits (10 μg/kg), and infant formula (0.5 μg/kg) (Chen et al., 2018; Li et al., 2021). Besides, Brazil has applied 10 μg/kg OTA standards to barley, legumes, rice, and maize; while Israel and Uruguay have set OTA standards of 50 μg/kg in pulses, crops, coffee and cereals. In USA, Food and Drug Administration (FDA) has not set advisory limits or action levels for OTA in any commodity. China has also set the legal limits of OTA in grapes, wine and must (2 μg/kg), grains, beans, nuts, coffee beans and their products (5 μg/kg) as well as instant coffee (10 μg/kg) (GB, 2017). Meanwhile, for OTA contamination has cause serious economic losses for manufacturers and exporters, the development of efficient techniques for OTA removal or reduction its toxicity in food raw material and products has become a focus of investigation (Gonçalves et al., 2019).

According to the present reports, various different ways called as physical, chemical and biological approaches are applied for the degradation or removal of OTA. Among them, some physical and chemical strategies have been used in food processing and achieved some success in the field of food and feed industries (X. Luo et al., 2020; K. Yang et al., 2020). A number of new techniques are trying to reduce the risk of OTA contamination. For example, cold plasma and pulsed light have received great attention (Casas-Junco et al., 2019; Hoppanova et al., 2020). At the same time, a large number of researchers have conducted the OTA control based on microorganisms from different sources and revealed their mechanisms (Hashemi et al., 2021). Although these microbial approaches are mostly carried out in laboratory scale, they are especially outstanding in the nutritional quality, sensory properties, high safety and detoxification efficacy. The generation and application of enzymes from different microorganisms have also received great attention (Leitao and Enguita, 2021).

At present, some literatures have reviewed the researches of OTA in foods. These studies mainly focused on the biosynthesis of OTA, DNA damage and harmful to people's health, occurrence and contamination in different kinds of food, detection and quantitative analysis, control of OTA and its producing strains as well as the fate of OTA in ruminants (Chen et al., 2018; Li et al., 2021; Sadiq et al., 2019). This study principally summarizes the current research in the removal, degradation and toxicity reduction of OTA by microbial approaches, including the latest OTA control techniques, the merits and shortcomings of various methods, metabolic pathways, metabolites and their safety.

## 2 Control of OTA contamination by biological approaches

In view of the advantages of better safety, availability, flavor and nutritional quality, many scientists have focus on the degradation (Table 1) or adsorption (Table 2) of OTA by the biological methods. At present, a series of microorganisms (such as yeast, bacteria and fungi) and enzymes have been applied for OTA elimination.

**Table 1:** The degradation of OTA by various microorganisms.

Species/strains	Source	Medium	Reaction conditions	Microorganism amount (Log CFU/mL)	Concentration of OTA (μg/mL)	Degradation rate (%)	Products	Reference
*Kloeckera lindneri*, *Metschnikowia pulcherrima*, *Rhodococcus erythropolis*, *Pichia guilliermondii*	a	PM broth (yeast extract 0.5%, sucrose 1%, peptone 0.5% and malt extract 0.2%)	30 °C, 15 d	6–8	7.5	25.8–84	NR	Patharajan et al. (2011)
*Phaffia rhodozyma*	Astaxanthin producing yeast isolates	PM broth	20 °C, 15 d	6	7.5	more than 90	OTα	Zhao et al. (2020)
*Metschnikowia pulcherrima, Lactobacillus rhamnosus*	Tempranillo winegrapes	Wine, TSB medium	pH 3.5, pH 6.5	8	1	34, 55	OTα	Minguez et al. (2020)
*Bacillus subtilis CW14*	fresh elk	LB medium	30 °C, 24 h	–	1	71.3	NR	Xu et al. (2021)
*Yarrowia lipolytica* Y-2	The surface of grapes	PM broth	In a shaker at 180 rpm, 28 °C	8	1	97.2	OTα	Zhang et al. (2018)
*Yarrowia lipolytica* Y-2; *Brevundimonas vesicularis*	Vineyard	Water	28 °C	7, 8	1	84; 100	NR	Wang et al. (2014)
*Yarrowia lipolytica* Y-2	Lactosan vineyard	Polytoma medium	In a shaker at 180 rpm, 28 °C, pH 4.0	8	0.1, 1, 2	88–95.7	NR	Yang et al. (2016)
*Pediococcus parvulus*	Douro wines	MRS medium	30 °C, 2 d	5	25	100	OTα	(Zhang et al., 2017)
*Acinetobacter calcoaceticus*	Soil samples	Minimal Medium Peptone	24 °C, 6 d	–	10	82–91	OTα	Peng et al. (2022)
*Lysobacter* sp. *CW239*	Soil samples	ME medium	37 °C, 24 h	–	0.02–0.1	86.2	OTα	Wei et al. (2020)
*Alcaligenes faecalis*	a	Luria-Bertani medium	30 °C, 48 h, 220 rpm	–	1	92	OTα	Zhang et al. (2017)
*Bacillus amyloliquefaciens* ASAG1 a	Grain depot-stored maize	No. 4 nutrient culture media	31 °C, 10 h	–	1	98.5	OTα	Chang et al. (2015)
*Aspergillus niger W-35*	Cereals	Commercial feeds	37 °C, 12 h	6	2	37	OTα	Zhao et al. (2020)
*Bacillus subtilis* CW 14; CW 14 a	Fresh elk droppings collected from Beijing Zoo	Luria–Bertani nutrient broth	37 °C, 200 rpm, 24 h	7	6	47.1; 97.6	NR	Shi et al. (2014)
*Bifidobacterium adolescentis*, *Bf. bifidum*, *Bf. breve*, *Bf. longum*, *Lactobacillus casei*, *L. delbrueckii bulgaricus*, *L. johnsoni*i, *L. paracasei*, *L. plantarum*, *L. rhamnosus*, *L. salivarius*, *L. mesenteroides*	a	MRS broth	37 °C, 24 h	8	0.6	29.6–99	OTα	Luz et al. (2018)
*Brevibacterium casei*, *B. linens*, *B. iodinum*	a	BSM	30 °C	–	0.011	100	OTα	Rodriguez et al. (2011)
*Cupriavidus basilensis* ŐR16	a	LB medium	28 °C, 72 h	–	20	100	OTα	Ferenczi et al. (2014)
*Brevundimonas naejangsanensis*	Soil	LB medium	37 °C, 24 h	–	1	100	OTα	Peng et al. (2022)
*Lactobacillus acidophilus*	a	MRS broth	37 °C	9	5	96	NR	Fuchs et al. (2008)
*Pediococcus parvulus* UTAD 473	Red wines of the Douro region	MRS broth	30 °C, 7 d	9	1	90	OTα	Abrunhosa et al. (2014)
*Rhodococcus erythropolis* GD2A, BRB 1AB; *Rhodococcus pyridinivorans* K402, K408	Natural soil, oil contaminated soil	LB medium (10 g tryptone, 5 g yeast extract, 9 g NaCl, pH 7,0)	28 °C, 72 h, 170 rpm	–	0.002	13.93–34.01	NR	Cserháti et al. (2013)
*Aspergillus. niger*, *A. carbonarius*, *A. fumigatus*, *A. clavatus*, *A. ochraceu*s, *A. versicolor*, *A. wentii*, *Cladosporium* sp., *Penicillium aurantiogriseum*, *Penicillium spinulosum*	Portuguese grape	Yeast extract sucrose medium: 2% of yeast extract from Difco,15% of sucrose	25 °C, 6 d	–	1	>80	OTα	Abrunhosa et al. (2002)
*Aspergillus carbonarius* SA332, *A.niger* GX312, *A. Japonicus* AX35	French grapes	Modified yeast extract broth medium and synthetic grape juice medium	240 rpm, 25 °C, 12 d	6	2	45–99	OTα	Khalil et al. (2021)
*Aspergillus*	Soil sample	MEA culture medium	30 °C	6	10	30–99	OTα	Xiong et al. (2017)
*Aspergillus tubingensis*	Traditional Korean meju	Soytone-Czapek medium	25 °C with shaking at 100 rpm, 14 d	6	0.04	more than 90 at pH 5, and 75.3–80.3 at pH 7	OTα	Cho et al. (2016)
*Aureobasidium pullulans*	a	Lilly-Barnett medium	23 °C, 160 rmp, 6 d	8	0.8	75–90.5	OTα	De Felice et al. (2008)
*Aspergillus oryzae*	Soil	PDA medium	30 °C, 72h	6	10	94	OTα	Xiong et al. (2021)
*A. carbonarius 10614, A. niger 10443*	Wine grapes	Wine	25 °C, 7 d	6	0.24	83.44	OTβ, OTC etc.	Freire et al. (2020)
*Aspergillus niger*	Chinese fermented soybean	PDB medium	28 °C, 48 h	4.30	1	89.40	OTα etc.	(Zou et al., 2022)
*Cryptococcus podzolicus*	a	PM medium	28 °C, 5 d	8.00	1	100	OTα	(Wei et al., 2020)
*Trichoderma afroharzianum*	a	0.9% NaCl	37 °C, 8 d	8.00	0.01, 0.1, and 1	31–46	NR	(Dini et al., 2022)
*Lactobacillus acidophilus*	Human urine	MRS/PBS	37 °C, 48 h	5–6	1	≤15%	NR	(Ragoubi et al., 2021)

a These strains were provided by professional organization or University, but the original separation information of them is not expressed in the original text; -- Not provided in the original text; NR, Not Report.

**Table 2:** The adsorption and removal OTA by various microorganisms.

Species/strains	Source	Medium	Reaction conditions	Microorganism amount (Log CFU/mL)	Concentration of OTA (μg/mL)	Removal rate (%)	Status	Reference
*Saccharomyces cerevisiae*	a	White grape and blackcurrant juice	30 °C, 24 h, static conditions	6	1	82.8–85.1 in grape and 10.7–65.2 in blackcurrant mediu	dead	Piotrowska et al. (2013).
*Candida famata, Candida guilliermondii, Candida lusitaniae, Cryptococcuslaurentii, Kloeckera* spp.*, Rhodotorula glutinis*	Different wine-grapes of Turkey	Phosphate-buffered saline (PBS) and white wine	25 °C, 4 h	8	1	1.96–26.11/4.1–31.31	viable/dead	Var et al. (2009)
*Candida intermedia*	a	Grape juice	25 °C, 48 h, 100 rpm, in the dark	9	20	>80	immobilized yeast cells	Farbo et al. (2016)
*Debaryomyces hansenii*	a	YMB medium	28 °C, 24 h, shaking (300 rpm)	5.3	7	>98	viable/dead	Gil-Serna et al. (2011)
*Phaffia rhodozyma*	–	PM broth (0.5% yeast extract, 1% sucrose, 0.5% peptone and 0.2% malt extract)	20 °C, 15 d	6	7.5	23/45	viable/dead	Zhao et al. (2020)
*Saccharomyces cerevisiae*	a	Red, rose and white must samples	12 °C, 90 d	–	0.01, 0.5, 1.0, 2.0 and 4.0	73–90	viable	Csutorás et al. (2013).
*Saccharomyces cerevisiae*	a	Wine	28 °C, 10 d	–	0.005	34.52–48.96	viable	Pulvirenti et al. (2020)
*Kefir grains (Lactobacillus kefiri*, *Kazachstania servazzii*, *Acetobacter syzygii)*	a	Milk	Aerobically, 25 °C, 24 h	7–11	1	15–81	viable	Taheur et al. (2017).
*Saccharomyces cerevisiae*	Grapes	Grapes must	25 °C	6	2000	6.42–8.59	viable	Petruzzi et al. (2017)
*Saccharomyces cerevisiae* BS	a	PBS (pH 7.2)	Static conditions or shaking, 30 °C for 24 h	–	1	75/77	viable/dead	Piotrowska (2012)
*Saccharomyces cerevisiae* (EC1118)	a	Model wine	214 h	–	1000	28.7/94.9	viable/dead	Nunez et al. (2008)
*Hanseniaspora uvarum U1*	grapes	PDB broth	30 °C for 48 h	3	0.5, 1	46.27–82.96/24.23–78.64	viable/dead	Gomez-Albarran et al. (2021)
*Saccharomyces cerevisiae*, *S. bayanus*	a	Yeast peptone glucose, synthetic grape juice and natural grape juice	30 °C, 2 h	7	2	34-45/17-75	viable/dead	Bejaoui et al. (2004)
*Saccharomyces cerevisiae* (RC008, 009, 012, 016)	a	YPD broth	37 °C, 1 h, shaking	7	1, 5, 10, 40, 100	14.5–74.2	viable	Armando et al. (2012)
*Saccharomyces cerevisiae* (RC212, BM45, W13, W47, Y28)	a	Model wine buﬀer	25 or 30 °C, 9 d, under dynamic conditions	–	2000	2.47–81.87	viable	Petruzzi et al. (2014b)
*Bacillus subtilis*	Kimchi	Wine	35 °C, 6 h	7–8	0.02	78.58	viable	Shukla et al. (2020)
*Saccharomyces cerevisiae* (TP5, TT173)	Native microflora of wine fermentations	Pasteurized must (pH 3.40, 20 °brix)	25 °C	–	0.0041	78.57–100/46.87–85.71	viable, cell walls/cells	Caridi et al. (2012)
*Saccharomyces cerevisiae* (W13, W28,W46)	Uva di Troia grape	Sugar 200 g/L; sugar 200 g/L + DAP; sugar 250 g/L; sugar 250 g/L + DAP	25 and 30 °C, without shaking	6	2	6-70, depending on environmental conditions	viable	(Petruzzi et al., 2014a)
*Saccharomyces cerevisiae* (W13, W28, W46, W47, Y28, Y20, W40)	Five wild strains	Grape must	25 or 30 °C. without shaking	6	2	20.34–53.79	viable	Petruzzi et al. (2015)
*Cyberlindnera jadinii, Candida friedrichii, Candida intermedia and Lachancea thermotolerans*	a	Commercial grape juice	25 °C, 8 d, agitation (100 rpm), in the dark	6.3	0.02	20–67.5	dead	Fiori et al. (2014)
*Saccharomyces cerevisiae*	a	Phosphate buffer	25 °C,1 h	4	0.1	82–91	Yeast cell wall	Ul Hassan et al. (2021)
*Lactobacillus plantarum*, *L. brevis LOCK*, *L. sanfranciscensis*	a	MRS medium and PBS buffer	30 °C, 24 h	4	1	14.64–35.01/46.29–59.82	viable/dead	Piotrowska (2014)
*Lactobacillus plantarum*, *Lactobacillus brevis*, *Leuconostoc mesenteroides*, *Pediococcus acidilactici, Oenococcus oeni*	a	Basal medium	30 °C, 48 h	–	0.004	8.23–28.09	viable	Muhialdin et al. (2020)
*B. subtilis*	a	Nutrient broth	35 °C, 48 h, 150 rpm, in the dark	–	0.04	22/45	viable/dead	Shukla et al. (2018)
*Lactiplantibacillus plantarum*	Brazilian artisanal cheeses	Potassium phosphate buffer *in vitro*	37 °C for 15 min, pH = 3.0	8	1	30–80	dead	Moller et al. (2021)
*Lactobacillus acidophilus*	Human urine	MRS/PBS	37 °C, 48 h	5–6	1	≤15%	NR	(Ragoubi et al., 2021)

a These strains were provided by professional organization or University, but the original separation information of them is not expressed in the original text; -- Not provided in the original text.

### 2.1 Reduction of OTA contamination by yeast

Fermentation is one of the most important forms of food processing. Decreasing the contamination of OTA by yeasts has been always the research hotspot. Now, various kinds of yeasts have been designed to reduce OTA content in musts, wines and others (M. Zhao et al., 2020). *Saccharomyces cerevisiae* are the most widely studied species. It has been reported that 20.34–76.44% of OTA can be reduced by genetically distinct strains of *S. cerevisiae*, while that for the strains of *Cyberlindnera jadinii*, *Candida friedrichii*, *Candida intermedia* and *Lachancea thermotolerans* were 20–67.5%. All of OTA (200 ng/mL) in fermentation broth can be degraded by *Trichosporon mycotoxinivorans* (Ortiz-Villeda et al., 2021; Petruzzi et al., 2015). In many cases, the decrease of OTA content by yeast can be divided into biodegradation and bio-adsorption.

**Fig. 1:**
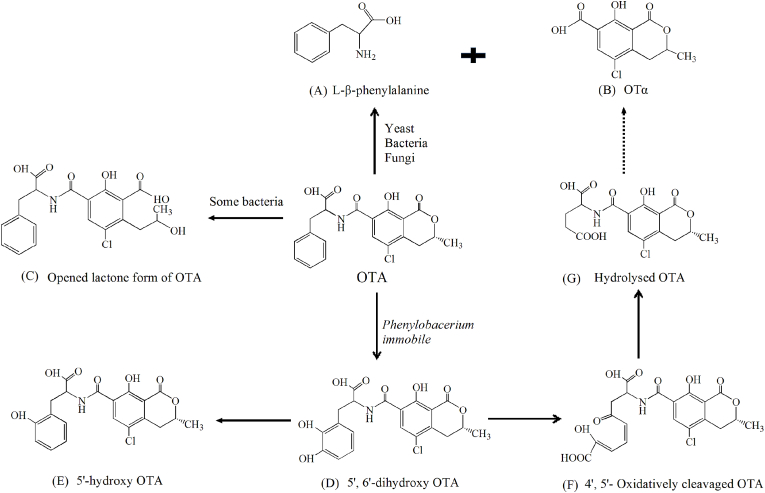
The main biodegradation pathways and metabolites of OTA.

At present, OTA degradation is mainly conducted by the hydrolysis of amide bond between the iso-coumarin residue and phenylalanine by carboxypeptidase. The major degradation products are the amino acid L-β-phenylalanine (Fig. 1A) and OTα (B). The toxicity of them is significantly less than that of OTA (W. Wei et al., 2020). However, OTα might induce the exchange of sister chromatid at high concentrations (Föllmann et al., 1995). The degradation processes depend on the types of strains, biomass concentration and environmental conditions. For example, more than 80% of OTA can be significantly eliminated by *Yarrowia lipolytica* (10^8^ cells/mL) at 28 °C and a pH value of 4. The increasing of cells amount and decreasing of pH values (4–7), temperature as well as toxin concentration were beneficial for OTA degradation (Q. Yang et al., 2016). Abrunhosa et al. (2014) proved that OTA can be fully converted into OTα in 6 days and 2 days with the increasing of cells biomass ranging from 10^3^ to 10^9^ CFU/mL. However, in some case, the expected by-products of phenylalanine and OTα were not detected either in the media or pellet (Patharajan et al., 2011). This may be caused that these two kinds of products can react with various compounds in the media. On the other side, there may be another degradation pathway, which should be further confirmed.

Adsorption is an effective microbial method for OTA removal. This process can be done not only by inactivate cells, but also living cells. A growing number of studies have found that a small amount of binding OTA could be released back into the culture and toxin adsorption is partially reversible. M. Piotrowska (2012) indicated that 11–22% of initially binding OTA could be recovered from the *S. cerevisiae* cells by washing three times using phosphate-buffered saline. In addition, the dead cells displayed better properties than that of living cells for OTA removal. It has been reported that 14.8–35% of OTA in medium can be removed using live bacterial cells, while that for the thermally inactivated cells was 46.2–59.8%. Furthermore, the reduction rate of pretreated cells (with cell wall partially removed) was from 3.9% to 5.6%, and protoplasts (cells without cell wall) had no ability for OTA adsorption (Małgorzata Piotrowska, 2014). The results demonstrated that the adsorption capacity of OTA depended on the cell wall structure. The cell walls of yeast were composed by two layers. The inner layer consisted of β-glucan and provides cell walls strength, while the outer layer was made up of mannoproteins and the glycan portion of them was made of acidic and neutral oligosaccharides. Most of mannoproteins were covalently linked to the inner glucan layer and played important effects on the surface properties of cell. Because these strains presented different amount of mannoproteins, the adsorption capacity of them varied from each other (Ortiz-Villeda et al., 2021). Huwig et al., (2001) proposed that cell walls contained a series of polysaccharides (such as β-glucans, mannan), proteins and lipids, which supplied lots of adsorption sites for OTA binding. Suvi Vartiainen et al. (2020) proved that OTA could be markedly removed by yeast cell wall extracts (YCWE) and the efficiency depended on the pH value and digestive physiological conditions. The results indicated that, when the pH value was lower than 6.5, the binding efficiency of OTA to YCWE was effectively enhanced; while an opposite phenomenon was observed with the pH value more than 6.5. The altering of pH value can lead to conformational changes of OTA molecule, β-D-glucans as well as the bioactive component of yeast cell wall extracts, which may affect the geometry of binding site and affinity of interaction. The mechanisms mainly include ionic interactions, hydrophobic interactions and hydrogen bonding. It has been also demonstrated that ionic interactions and electrostatic interactions took important role in OTA adsorption. The parietal mannoproteins was the main binding sites (Caridi et al., 2012).

### 2.2 Reduction of OTA contamination by bacteria

Many types of bacteria play an important role in OTA control. A variety of bacteria, such as *Acinetobacter*, *Alcaligenes faecalis*, *Acinetobacter* sp. *neg1*, *Bacillus amyloliquefaciens*, *Bacillus licheniformis*, *Brevibacterium* spp., *Enterobacter*, *Lactobacillus plantarum*, *L. brevis*, *L. sanfrancisco*, *Pantoea*, *Brevundimonas naejangsanensis* isolated from fermented food Kimchi, soybean, wines or soil samples exhibit great OTA control ability (Abrunhosa et al., 2014; Peng et al., 2022; Wei et al., 2019). The metabolites are OTα and phenylalanine, which indicates that these degradation processing is similar to that of yeast. Campos-Avelar et al. (2020) displayed that 33 strains of actinobacteria can completely degrade OTA in liquid medium while 5 strains can be used for decrease OTA content on solid medium. However, none of the common degradation products of OTα or L-β-phenylalanine were found, which indicated that there may be different degradation mechanisms. Besides, the degradation of OTA can be conducted via the hydrolysis of the lactone ring (Fig. 1C). In this case, the lactone bond of OTA was hydrolysed by esterases or lactonohydrolases from microbial cells, such as *Acinetobacter calcoaceticus, Agrobacterium tumefaciens, Fusarium oxysporum* and others. As with alkaline treatment, the degradation product was an opened lactone form of OTA with the similar toxicity to rats as that of OTA, as well as less toxic to mice and *Bacillus brevis.* However, the lactone ring opening of OTA was reversible and the toxin can be regenerated under acidic conditions (Karlovsky, 1999; Shimizu et al., 2001). Wegst and Lingens (1983) also reported that OTA might be detoxified by *Phenylobacerium immobile* at 25 °C in 3–5 h with different pathways and metabolites (Fig. 1). (i) Attacking on phenyl moiety by Ddioxygenase and forming of 5′, 6′-dihydroxy OTA (D); (ii) The spontaneous dehydration reaction of the dihydrodiol derivative and obtaining of 5′-hydroxy OTA (E); (iii) The meta-proximal cleavage of the catechol moiety and the formation of 4′, 5′- Oxidatively cleavage OTA (F); the generation of hydrolysed OTA (G) by a hydrolytic cleavage as well as the releasing of OTα.

In addition, many bacteria also exhibit excellent adsorption of OTA, especially lactic acid bacteria. It has been reported that several kinds of lactic acid bacteria (such as *Lactobacillus brevis*, *L. sanfranciscensis*, *O. oeni*, *L. rhamnosus, Pediococcus parvulus* et al.) showed good performance for OTA removal, which depend on the cell walls (Muhialdin et al., 2020). The cell walls of lactic acid bacteria were composed of a peptidoglycan matrix, a proteinaceous S layer and neutral polysaccharide. Peptidoglycan matrix was the main component of cell wall and contains other components such as lipoteichoic acids and teichoic acids (Leitao and Enguita, 2021). Especially, teichoic acid may account for more than 50% of cell wall weight and appeared to be primarily responsible for its hydrophobicity (Delcour et al., 1999). According to Małgorzata Piotrowska (2014), the surface of lactic acid bacteria was hydrophilic and these cells were strong electron donors and weak electron acceptors. The binding of OTA was mainly conducted by the hydrophobic nature and cell wall components, such as polysaccharides, peptidoglycan, lipoteichoic acids and teichoic (Shukla et al., 2018). Haskard et al., (2000) also concluded that hydrophobic interaction played an essential role in the binding process, while electrostatic repulsion had a minor effect. The removal of OTA was also affected by electron donor-acceptor, Lewis acid-base interactions as well as S-layer protein. Besides, the ethyl methanesulfonate-induced mutagenesis can enhance the OTA binding efficiency of some lactic acid bacteria (Sadiq et al., 2019). At the same time, the heat-treated cells of *Bacillus subtilis* can be used for removal of OTA (Shukla et al., 2018). This is caused that the surfaces of *B. subtilis* have abundant peptidoglycan. Based on the specific amino acid sequence of the peptide bridges between N-acetylmuramic acid chains, peptidoglycan played key role in OTA adsorption (Niderkorn et al., 2009).

### 2.3 Control the OTA contamination by fungi

**Fig. 2:**
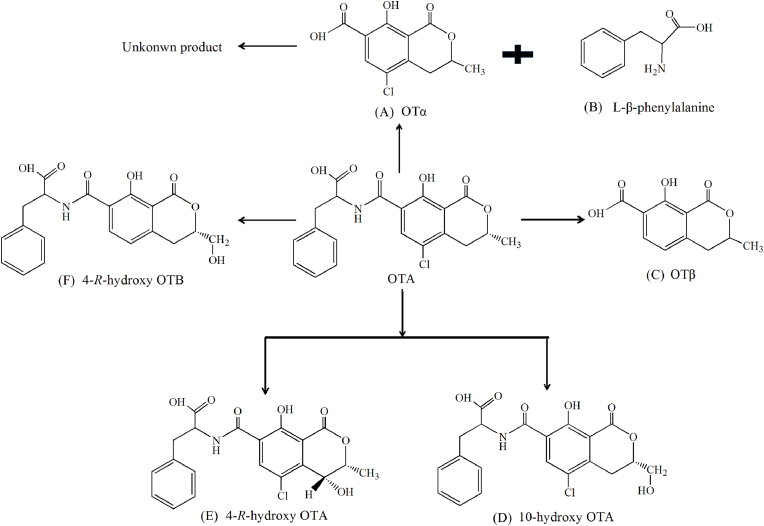
The degradation pathways and metabolites of OTA by some special fung.

Fungi are one of the important bacterial resources for OTA degradation. About 250 fungi species isolated from Korean meju or grapes were used for OTA biodegradation. These strains, such as *Aspergillus. niger*, *A. carbonarius*, *A. japonicas*, *A. wentii*, *A. clavatus*, *A. ochraceus*, *A. pullulans*, *A. versicolor*, *Alternaria*, *Botrytis*, *Cladosporium*, *Penicillium,* exhibited different OTA degradation capabilities with the metabolites of OTα and phenylalanine (Abrunhosa et al., 2002; Cho et al., 2016). The results showed that most fungi strains had the same degradation pathways and products as that of yeast and bacteria. However, some strains display special characteristics for OTA degradation. *Aspergillus* exhibited the fastest degradation rate than others and the product of OTα can be further decomposed as an unknown compound (Khalil et al., 2021; K. Xiong et al., 2021). The species of *A. ochraceus* and *A. wentii* can produce different metabolites, which indicated that the degradation pathway of OTA may be differ from others (Abrunhosa et al., 2002). Xiao, Marquardt, Abramson, and Frohlich (1996) isolated metabolites of OTα (A), L-β-phenylalanine (B), OTβ (C), 10-hydroxy OTA (D), 4-*R*-hydroxy OTA (E) and 4-*R*-hydroxy OTB (F) from the culture of *A. ochraceus* (Fig. 2)*.* It has been further proved that OTα and 4-*R*-OH OA were the main metabolites and can be consistently produced by the fungal culture, while the generation of 10-OH OA was conditional. At the same time, similar metabolites of OTA are also proved in the urine of rats. The results indicated that some fungal systems and animal had similar enzyme systems, which mainly dominated the hydrolysis and hydroxylation of OTA (Gu et al., 2021).

A large number of studies have shown that the elimination of OTA using fungi is mainly conducted by their degradation. In this process, the viable cells play more important role than that of dead cells. In addition, fungi can be also used for OTA adsorption. The adsorption of OTA by fungi is mainly due to hydrophobicity or the negative charge. For example, *Botrytis fabae* conidia were hydrophobicity due to the action of hydrophobins or lipids, while *A. fumigatus* conidia had a large amount of negatively charged carbohydrates. These properties are beneficial for the adsorption of living or dead conidia. Binding of OTA onto the surface of black aspergilla conidia is related to hydrophobic interactions. Besides, OTA molecules could be ionized in acidic media and then be highly reactive with other negatively charged molecules of fungal spores (Abrunhosa et al., 2014; Wasylnka and Moore, 2000).

### 2.4 Degradation of OTA by enzymes

In fact, the detoxification of OTA by microorganism is mainly conducted by the function of enzyme. As mentioned above, a large number of studies have shown that OTA can be mainly detoxified via the hydrolysis of carboxypeptidase from various microorganisms with the metabolites of OTα and phenylalanine. Some study further display that the degradation process is mainly associated with carboxypeptidase A and carboxypeptidase Y enzyme. The primary difference of them is the using of zinc ion within the protein for hydrolysis of the peptide at the C-terminal of the amino acid (Schrenk et al., 2020). Besides, a variety of enzymes such as lipases, laccase, amidases, deoxygenases, ochratoxinase and several commercial proteases are also able to conduct this reaction. Multifunctional recombinant enzyme based on zearalenone hydrolase and carboxypeptidase is generated to degrade OTA (Loi et al., 2018; Taheur et al., 2019). Luz, Ferrer, Mañes, and Meca (2018) suggested that the hydrolysis reaction could be mediated by carboxypeptidase A enzyme from the bovine pancreas, OTA-hydrolytic enzyme, commercial proteases and lipase as well as carboxypeptidase Y from *S. cerevisiae*. About 27.0% and 4.8% of OTA in model solution and beer can be degraded by peroxidase enzyme from *Armoracia rusticana* under the optimized conditions (pH 7, 30 °C, 25 mM ionic strength, 26 mM H_2_O_2_, 1 mM potassium ion) (de Oliveira Garcia et al., 2020).

Fanelli et al. (2015) isolated and sequenced the genome of a *Acinetobacter* sp. *neg*, which was able to degrade OTA via the reaction of peptide bond hydrolysis. In this process, β-metallo-lactamase is involved in the degradation of OTA. Although the intermediates of each enzyme may be different, the end product of them is always OTα. In addition, the degradation efficiency of OTA depends on enzyme activity, temperatures and their source. It has been showed that optimal temperatures of carboxypeptidase enzymes are 30 °C or higher, which might hamper their practical applications (Abrunhosa et al., 2014). A later study also confirms that 97.2% OTA can be degraded by intracellular enzymes within 4 h, indicating that this process was mainly due to the function of intracellular enzymes rather than extracellular enzymes (X. Zhang, et al., 2018).

### 2.5 Effects of different factors on OTA adsorption and removal

Based on the structural characteristics and surface properties of different microbial cells, it is easy to understand why the inactivate cells have good performance. For example, the OTA adsorption via living yeast cells is mainly achieved by the presence of hydrophobic pockets on the surface, while the pretreatment of heating or acid may facilitate the formation of more binding sites on the surface of inactivated cells than viable cells (Małgorzata Piotrowska, 2014). Heating may lead to the formation of Maillard reaction products, denaturation of proteins or pore generation, while acid media may be conducive to the release of monomers and further promote the break of polysaccharide chains into aldehydes (Bejaoui et al., 2004). On the other side, the physical and chemical characteristics of cell walls, such as the chemical composition, surface properties, losing or not of outer membrane barrier, thickness of the peptidoglycan as well as the environmental conditions, have significant effect on OTA adsorption (M Piotrowska, 2012). For the cell walls amount are related to the cell diameters and cell wall thickness, the relationship of them are applied to evaluate the number of cell walls. Małgorzata Piotrowska (2014) found that the yeast-OTA interaction was more of ‘adsorption type’. The strains with more cell wall content exhibit greater ability for OTA removal. Other studies also prove the correlation between the number of cell walls and toxin removal ability. The increasing of cells concentration would enhance the OTA adsorption. Zhao et al. (M. Zhao et al., 2020) suggested cells concentration was positively associated with OTA removal capacity of dead cells, but had no effect on OTA binding efficiency of living cells. Besides, the increasing of sugar and temperature are conductive to the improvement of OTA removal rate, and the addition of diammonium phosphate can further enhance this phenomenon (Petruzzi et al., 2015). This is caused that the OTA removal depends on the surface properties of yeast cells, which is affected by the growth conditions, such as nutrient availability and culture methods (Bejaoui et al., 2004). OTA reduction is also related to pH, temperature and ethanol content. At lower pH value, the greater ability of OTA removal is achieved, which is associated with the amino group ionization of OTA molecules in acidic media. The increasing OTA removal efficiency at higher temperature may be related to the release of cell wall polysaccharides under thermal stress (Bejaoui et al., 2004; Y. Luo et al., 2020). Besides, the OTA removal is also affected by ethanol concentration. It has been showed that 36–42% of OTA (2 μg/L) could be reduced by *S. cerevisiae* in cultures containing 100 g/L ethanol, while the maximum reduction rate of OTA by *Oenococcus oeni* is observed in cultures containing 5% ethanol. The present of ethanol is beneficial for the release of polysaccharides and mannoproteins by viable yeast cells as well as the promoting of cell autolysis. Meanwhile, the solubility of OTA may be also enhanced by ethanol at the acidic pH of the medium, so OTA in acidic ethanol beverages (such as wine) cannot be completely eliminated (Freire et al., 2020).

### 2.6 Evaluation the safety of biotransformation metabolites

As mentioned above, the safety of the reported degradation products is clear. In fact, due to the diversity of various microbial systems, the OTA degradation products are very complex. If the metabolites cannot be confirmed, the safety of them can be evaluated by cell tests or animal experiment. Yang et al. (2016) evaluated the safety of metabolic products using HepG2 cells. The testing results indicated that the toxicity of the biodegradation products was notably less than that of OTA. Zhao et al. (Zhao et al., 2021) verified the cytotoxicity of OTA degradation products with human kidney epithelial cells. Besides, zebrafish embryo model is applied to assess the teratogenicity and safety of OTA and OTα, the results indicate that hydrolysis is a reliable means of OTA detoxification (Csenki et al., 2019). It also confirms that this model is an effective tool to evaluate the safety of OTA metabolites. In addition, assessment the interaction between toxin transformation metabolites and gut microbes, gastrointestinal digestion or blood levels is very crucial (Luz et al., 2018; Xia et al., 2021).

## 3 The shortcomings of various methods

For a long time, a serial of methods have been explored for control the contamination of OTA in foods. Although these techniques mentioned above can detoxify OTA effectively, they still have some flaws and should be further improved.

The processing stages, such as alcoholic fermentation, malolactic fermentation, racking, fining and storage of wine, and the washing, scouring, milling as well as cooking in grape products, can effectively eliminate OTA content in food (Ortiz-Villeda et al., 2021; Osborne et al., 1996; J. Zhang et al., 2022). Despite all this, the reduction OTA contamination by these methods is mainly achieved by removal, rather than reducing its toxicity. Although these methods are beneficial for reducing OTA content in food system, they can also cause secondary contamination. In addition, different methods are usually used together to improve their efficiency for OTA control during food processing. Heating is a traditional way of food processing and plays an important role in food sterilization and preservation. Due to the stability, the temperature for OTA destruction is particularly high. It has been reported that only 20% of OTA can be degraded by heating at 100 °C for 160 min or 150 °C for 32 min. Therefore, this method inevitably has a serious negative impact on food quality and can be only used in the processing of special foods such as coffee roasting (Aguilar-Alvarez et al., 2021).

UV is widely used in the sterilization of liquid food and exhibits good ability to detoxify toxins. However, for UV light is easily absorbed by many compositions, this treatment is susceptible to food ingredients and physicochemical parameters, such as phenols, organic acids, amino acid, inorganic matter, pH value and ethanol (Shanakhat et al., 2019). As an ideal non-thermal sterilization technique, EBI has been applied in the preservation of a variety of foods and has shown some potential in toxins degradation. The defects of this technology are that the degradation of OTA depends on the irradiation dose, matrix compositions, toxin concentration and so on. This process may also destroy the nutrients of food, such as vitamins, proteins, unsaturated fatty acids and probiotics (Luo et al., 2017; K. Yang et al., 2020). In addition, some newly developed techniques including the ultra-high pressure, cold plasma and pulsed light have display extensive prospects in food processing, but these techniques are rarely used for toxin degradation. The pathways and safety of metabolites are not revealed (Casas-Junco et al., 2019; Moreau et al., 2013). Therefore, the application of these techniques in the field of OTA reduction should be further studied.

The control of OTA by chemical treatment is mainly achieved under acidic or alkaline conditions with high temperatures. The advantages of these methods are simple operation, low cot and easy industrialization, while chemical reagents tend to affect the quality of food, such as sensory properties and nutritional value. It has been reported that chemical treatment can produce brown or black spot and damage the surface of pepper (Jalili and Jinap, 2012; Jalili et al., 2011). At the same time, due to chemical reagents may cause residues; the using of them has strict regulations. Some chemical agents can reduce toxin content, but they cannot be applied in food processing. As one kind of strong oxidant, ozone has a good bactericidal ability and is helpful for preserving and extending the shelf life of food. However, ozone is also a toxic gas. It should be used in a closed environment with strict dosage to avoid damaging the body (Akbar et al., 2020).

Biological approaches are now widely studied for mycotoxin control with the advantages of great efficiency, high specificity. The existing studies suggest that the control of OTA by yeast and lactic acid bacteria are mainly carried out via adsorption; while that for bacteria and fungi are primarily degradation. During the degradation, OTA can be converted into less toxic metabolites in mild reaction conditions, which is environmentally friendly and no damage to nutrients (Gu et al., 2021). The drawback of microbial approach is the long processing time, which may take several days for degradation or adsorption. On the other side, the growth and metabolism of microbial cells may affect the taste and flavor of the product, microbial degradation method can only be used in specific liquid foods, such as the production of fruit wine by yeast and the fermentation of fruit juice by lactic acid bacteria. Besides, most of them are now laboratory scale and need to be further enlarged.

## 4 Future prospects and application

In general, the ideal treatment for toxin control should be: (i) fast and efficient for the reduction of toxin content and toxicity; (ii) not create or cause new contamination; (iii) not change the process and affect product quality; (iv) easy to operate in large-scale and industrialization. Therefore, the control of OTA can be improved in the following aspects.

(1) Existing methods are mainly carried out in simple buffer systems or simulated food systems. The effects of processing and food ingredients on OTA control have been not involved. For example, there are many studies on the degradation or adsorption of OTA in wine by yeast, while the effect of polyphenols in grapes on OTA elimination is not clear. In the process of juice fermentation with lactic acid bacteria, few studies have revealed the relationship between nutrient components and OTA reduction. The future research should be carried out closely around the food processing. Not only analyze the metabolic pathway and safety of transformed products, but also systematically evaluate the interaction between food components and OTA detoxification.
(2) With the characteristics of high specificity, great efficiency and no impaction on product quality, enzymatic hydrolysis is a growing trend of toxin degradation. It can not only avoid the accumulation of toxins like adsorbents to pollute the environment, but also avoid the production of secondary metabolites like microbial fermentation. Now, it is essential to improve the enzyme yield through screening of high-producing strains and optimizing culture conditions, or generate stable enzymes through large scale production of gene engineering and protein engineering. At the same time, it is important to change the molecular structure of enzymes and enhance their applicability to different environments, which is beneficial to promote OTA degradation. The revealing of biological transformation pathway and the safety of products is also very significant.
(3) A growing number of researches are focusing on the removal of toxins by functional materials. The problem with these methods is that the adsorption efficiency is low and the adsorption process has no specificity. More importantly, these adsorbents are difficult to separate and may remain in food system. In this regard, the development of magnetic materials provides a new idea for OTA adsorption. The combination of adsorbent and magnetic material can not only improve the removal rate of toxin, but also separate the adsorbent quickly to avoid their contamination. For instance, in many cases, the reduction of OTA content in wine or fruit juice during fermentation is mainly achieved by the adsorption of yeast and lactic acid bacteria rather than degradation. Magnetic material modified microbial cells can be used for toxins removal effectively. The development of magnetic adsorbents should be focused on the improvement of efficiency and specificity of OTA removal as well as the separation of adsorbents to reduce their residue in sample system. The residue and safety of magnetic adsorbents should be also evaluated. Besides, the use of immobilized microbial cells for fermentation may be a promising attempt.

## 5 Conclusions

In view of OTA toxicity and high contamination frequency, a growing number of strategies have been conducted to detoxify OTA in food. Although the existing methods can effectively decrease OTA content in foods or reduce its toxicity, the control of OTA in future should be conducted in several aspects. Firstly, both of degradation and removal of OTA should be conducted closely around food ingredients and food processing. The interaction between OTA detoxification and food ingredients should be further revealed. Secondly, it will be significant for the efficient degradation of OTA through the purification and modification of specific enzymes. At the same time, the generation of functional magnetic adsorbents based on the biological cells is also an important aspect for adsorption and removal of OTA. Finally, the biological transformation pathway, the safety of products as well as the residue and safety of magnetic adsorbents in food medium should be evaluated. By this way, appropriate OTA control methods for different food samples systems will be established to ensure food safety and human health.

## CRediT authorship contribution statement

**Leran Wang:** were responsible for the correction, improvement and finalization of the manuscript. **Qi Wang:** were responsible for the correction, improvement and finalization of the manuscript. **Saiqun Wang:** carried out the literature review and wrote the manuscript. **Rui Cai:** carried out the literature review and wrote the manuscript. **Yahong Yuan:** were responsible for the correction, improvement and finalization of the manuscript. **Tianli Yue:** provided the writing ideas and writing focus of this manuscript. **Zhouli Wang:** carried out the literature review and wrote the manuscript.

## Declaration of competing interest

The authors declare that they have no known competing financial interests or personal relationships that could have appeared to influence the work reported in this paper.
